# Novel viral vectors utilizing intron splice-switching to activate genome rescue, expression and replication in targeted cells

**DOI:** 10.1186/1743-422X-8-243

**Published:** 2011-05-19

**Authors:** Liane Viru, Gregory Heller, Taavi Lehto, Kalle Pärn, Samir El Andaloussi, Ülo Langel, Andres Merits

**Affiliations:** 1 Institute of Technology, University of Tartu, 50411, Tartu, Estonia; 2 Department of Neurochemistry, Stockholm University, SE-106-92, Stockholm, Sweden; 3 Department of Laboratory Medicine, Karolinska Institutet, SE-141 86, Huddinge, Sweden

## Abstract

**Background:**

The outcome of virus infection depends from the precise coordination of viral gene expression and genome replication. The ability to control and regulate these processes is therefore important for analysis of infection process. Viruses are also useful tools in bio- and gene technology; they can efficiently kill cancer cells and trigger immune responses to tumors. However, the methods for constructing tissue- or cell-type specific viruses typically suffer from low target-cell specificity and a high risk of reversion. Therefore novel and universal methods of regulation of viral infection are also important for therapeutic application of virus-based systems.

**Methods:**

Aberrantly spliced introns were introduced into crucial gene-expression units of adenovirus vector and alphavirus DNA/RNA layered vectors and their effects on the viral gene expression, replication and/or the release of infectious genomes were studied in cell culture. Transfection of the cells with splice-switching oligonucleotides was used to correct the introduced functional defect(s).

**Results:**

It was demonstrated that viral gene expression, replication and/or the release of infectious genomes can be blocked by the introduction of aberrantly spliced introns. The insertion of such an intron into an adenovirus vector reduced the expression of the targeted gene more than fifty-fold. A similar insertion into an alphavirus DNA/RNA layered vector had a less dramatic effect; here, only the release of the infectious transcript was suppressed but not the subsequent replication and spread of the virus. However the insertion of two aberrantly spliced introns resulted in an over one hundred-fold reduction in the infectivity of the DNA/RNA layered vector. Furthermore, in both systems the observed effects could be reverted by the delivery of splice-switching oligonucleotide(s), which corrected the splicing defects.

**Conclusions:**

Splice-switch technology, originally developed for genetic disease therapy, can also be used to control gene expression of viral vectors. This approach represents a novel, universal and powerful method for controlling gene expression, replication, viral spread and, by extension, virus-induced cytotoxic effects and can be used both for basic studies of virus infection and in virus-based gene- and anti-cancer therapy.

## Background

Viruses that infect vertebrate cells are capable of recognizing host cells and executing gene expression, genome replication and virion formation. The ability to use and control these processes is at the origin of virus-based gene-technology applications, such as the delivery of transgenes or the use of viruses for vaccinal or vaccine-carrier purposes. Based on their inherent ability to kill cancer cells, a number of viruses have been tested as anticancer agents, including those with double- [1,2] and single-stranded DNA genomes [3] and viruses with double- [4], negative- [5] and positive-stranded [6] RNA genomes. Typically, these viruses can also infect normal cells; engineering specificity for cancer cells is therefore crucial. Several strategies have been tested for this purpose, e.g., the creation of p53-sensitive mutants [7], the alteration of viral receptor specificity [8,9], the use of cancer-specific promoters in recombinant viruses [10,11] and the regulation of gene expression in oncolytic viruses using cellular microRNAs [12,13]. The general shortcomings of such viral vectors are their specificity to only certain cancer types and an attenuated oncolytic potential. In addition, genetically modified viruses, especially those with RNA genomes, tend to revert, or compensate for the introduced changes, which are usually unfavorable for the infection cycle. Therefore, new approaches are needed that are applicable to different viral systems and allow for the possibility of combination with other regulation strategies without compromising the anticancer properties of the vector.

Adenoviruses (family *Adenoviridae*) are currently the most extensively used oncolytic DNA viruses. They have non-enveloped virions, linear double-stranded genomes and replicate in the nucleus of infected cells [14]. Adenovirus gene expression, replication, virion formation and cytotoxicity are dependent on the E1A proteins expressed via a constitutively active promoter [15,16]. In many engineered adenovirus vectors, including the human adenovirus 5-based Adeasy system [17], the E1A region has been deleted and, therefore, these vectors are not capable of replication unless the E1A proteins are co-expressed.

Among positive-strand RNA viruses, the alphaviruses (family *Togaviridae*), including Semliki Forest virus (SFV), represent one of the most promising candidates for vaccine and anticancer vectors [6]. Alphaviruses form cytoplasmic, membrane-associated replicase complexes [18,19] and encode for two polyprotein precursors: a nonstructural (ns) polyprotein (replicase), translated directly from genomic RNA, and a structural polyprotein, from subgenomic (SG) mRNA [20]. The alphavirus-based replication-competent vectors contain a complete viral genome and one or more foreign-protein-encoding sequences [6,21], whereas replicon vectors lack the region coding for viral structural proteins and are unable to spread [22]. Typically, the RNA genomes of alphavirus-based vectors are rescued from cloned infectious cDNA by *in vitro *transcription [23]. Alternatively, cDNA can be flanked with eukaryotic transcription sequences; here, rescue involves cellular transcription by RNA polymerase II and nuclear exit of the RNA product followed by translation and subsequent replication. These vectors are commonly known as DNA/RNA layered vectors [24].

With the exception of poxviruses, all DNA viruses use splicing for the expression of some of their mRNAs. Therefore, the discovery that splicing patterns can be regulated by antisense splice-switching oligonucleotides (SSOs) [25-28] created novel possibilities for regulating viral gene expression and replication. In contrast, positive-strand RNA viruses never utilize splicing; however, the insertion of introns into their cDNA sequences is often used for the construction of corresponding DNA/RNA layered vectors [29-34]. Thus, all these viral vectors can be engineered to contain aberrantly spliced introns that block the expression of correct gene products (DNA viruses) or the rescue of infectious RNAs (DNA/RNA layered vectors). Such introns frequently occur in nature; for example, human beta thalassemia is often caused by a single-nucleotide mutation within an intronic segment of the human beta-globin gene, creating an aberrant splicing site [35,36]. Targeting aberrant splicing sites with antisense SSOs has resulted in efficient correction of splicing in both *in vitro *[37,38] and *in vivo *models [39,40].

Here, we present evidence that insertion of aberrantly spliced introns into the genome of a recombinant adenovirus vector results in severely defective marker-gene expression. Similar manipulations of DNA/RNA layered SFV vectors also resulted in a reduction of the rescue of infectious transcripts. Both of these defects were reversed by SSOs. In essence, the presence of such introns in crucial regions of the viral vector represents a lethal mutation and SSOs represent an artificial and efficient cofactor required by the constructed vector. This approach represents a novel, universal and powerful method for controlling gene expression, replication, viral spread and, by extension, virus-induced cytotoxic effects.

## Methods

### Cells and media

HeLa cells were grown at 37°C with 5% CO_2 _in Iscove's modified Dulbecco's medium (IMDM; Invitrogen, Carlsbad, CA, USA) supplemented with 10% fetal calf serum (PAA, Pasching, Austria), 100 U/ml penicillin and 0.1 mg/ml streptomycin. HEK293 cells were grown in the same conditions but in Dulbecco's Modified Eagle Medium (DMEM; Invitrogen). BHK-21 cells were grown in Glasgow's Minimal Essential Medium (GMEM; Invitrogen) containing 7.5% fetal calf serum, 2% tryptose phosphate broth (TPB), 200 mM HEPES, 100 U/ml penicillin and 0.1 mg/ml streptomycin.

### Luciferase reporters and introns designed for insertion into DNA/RNA layered vectors

The luciferase reporter Luc7 contains a second intron from the human beta-globin gene with a thalassemic T705G mutation (IVS2^705^). Its sequence corresponds exactly to the reporter from the previously described pLuc/705 plasmid [38]. All other luciferase reporters were created by site-directed mutagenesis. To obtain LucWT, the T705G mutation was reverted; for Luc6+7, the mutation C654T (IVS2^654^) [36] was added; and for Luc0, the intron was deleted.

For the insertion of the wild-type (wt) or mutant introns (with C654T, T705G or C654T + T705G mutations) into SFV DNA/RNA layered vectors, the synthetic DNA fragments running from unique XbaI (6,637) to BglII (6,712) recognition sites were obtained from GeneArt AG (Regensburg, Germany) and inserted into SFV cDNA. Intron was positioned between nucleotides 6,696 and 6,697 (all nucleotide positions are given with respect to the sequence of an infectious cDNA clone designated pSFV4 [23]).

### Construction, propagation and use of adenovirus vectors containing luciferase reporter

Recombinant adenoviruses expressing firefly luciferase from reporter genes containing wt or mutant introns were constructed using the Adeasy system. The sequences of LucWT, Luc7 or Luc6+7 reporters were PCR amplified and cloned into the pShuttle-CMV vector using the restriction sites KpnI and NotI. Recombination with the Adeasy1 plasmid, transfection of HEK293 cells and rescue of the recombinant adenovirus was performed as previously described [17]. Adenovirus stocks were propagated by one passage in HEK293 cells; virus particles were collected, purified and titrated using an endpoint-dilution method.

For infection experiments, HeLa cells grown on a 24-well plate (with a growth area of 2 cm^2 ^per well) to 80% confluence were infected with virus stock at 0.5 PFU/cell in 200 μl serum-free IMDM media. Cells were incubated with the virus for 2 h at 37°C, after which 1 ml of complete IMDM media was added. Cells were incubated for an additional 24 h at 37°C, collected and lysed, and luciferase activity was then measured using a Luciferase assay system (Promega, Madison, WI, USA).

### Construction of SFV DNA/RNA layered replicon and replication-competent vectors

Recombinant SFV DNA/RNA layered-replicon and replication-competent vector plasmids were based on the previously described pCMV-SFV1 and pCMV-SFV4 [29], respectively. The luciferase gene with an intron was inserted into the region of the cDNA corresponding to the nsP3 gene of SFV [41]. In the case of vectors with two introns, the second intron was inserted into the cDNA region corresponding to the SFV nsP4 gene. If the primary transcript remains unspliced or is aberrantly spliced, these introns interrupt the reading frame encoding for viral replicase, resulting in a lethal defect. The combinations of inserted introns and the designations of the corresponding vectors are listed in Table 1.

**Table 1:** Combinations of wild-type (WT) and aberrantly spliced introns used in SFV DNA/RNA layered vectors.

Luc gene Intron in nsP4	Luc0	LucWT	Luc7	Luc6+7
None	nc	A1	A2	A3

WT	B0	B1	Nc	nc

C654T	C0	nc	C2	nc

T705G	D0	nc	D2	nc

C654C + T705G	E0	nc	Nc	E3

nc: vector not constructed.

**Figure 1:**
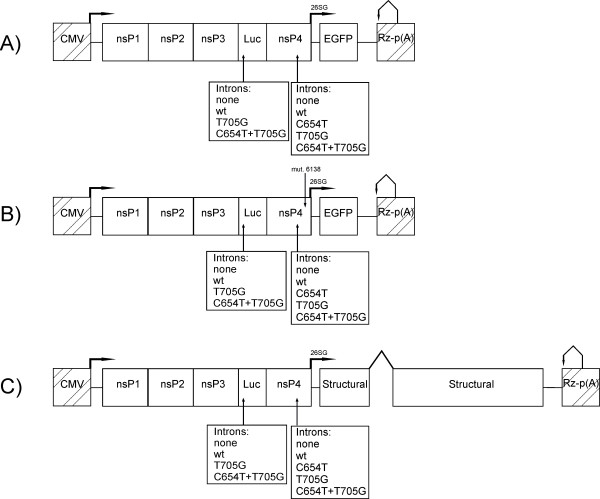
**Schematic presentation of the SFV DNA/RNA layered vectors**. The plasmid backbone of the vector is not shown; CMV, immediately early promoter of human cytomegalovirus; Rz, hepatitis delta virus negative strand ribozyme; p(A), simian virus 40 late polyadenylation signal. The nsP1-4, region corresponding to ns-proteins of SFV; Luc, coding sequence of the Luc reporter; 26SG, SG-promoter of SFV. Arrows in boldface indicate transcription initiation sites of the CMV and 26SG promoters; bent arrows indicate processing of mRNA transcript by ribozyme and lines indicate noncoding regions of SFV. Sites used for intron insertion are indicated by arrows; inserted introns and thalassemic mutations present in these introns are indicated under the drawings. DNA/RNA layered replicon vector without (A) or with (B) frame-shift (FS) mutation (indicated by arrow) in the region corresponding to the nsP4 gene. EGFP, coding sequence of EGFP reporter. (C) DNA/RNA layered replication-competent vector. "Structural" indicates the region corresponding to structural genes (C-E3-E2-6K-E1) of SFV; wt intron from rabbit beta-globin gene, inserted in this region, is shown by lines above drawing.

In DNA/RNA layered replicon vectors, the sequence encoding for EGFP was placed under the control of the SFV SG promoter so its expression was strictly dependent on the ability of the vector to initiate RNA replication and transcription in transfected cells. In addition, variants from all replicon vectors were prepared by the introduction of a frame-shift (FS) mutation (4 b insertion) into the replicase region (at position 6,138) of SFV. The designs of all the DNA/RNA layered vectors are shown in Figure 1 and the sequences of all vectors and details of their construction are available from the authors on request.

### Antisense oligonucleotides and transfection procedures

Phosphorothioate 2''-OMe RNA oligonucleotides designated as ON705 (5'-CCUCUUACCUCAGUUACA-3'), ON654 (5'-GCUAUUACCUUAACCCAG-3') and ONINV (5'-CCUCUUACACUCGUUACA-3') [42] were obtained from GE Healthcare (Little Chalfont, UK). If not otherwise stated, oligonucleotides were used in transfection mixtures at a final concentration of 100 nM (in cases where two oligonucleotides were used, each were used at a final concentration of 100 nM) and delivered to the cells using Lipofectamine™ 2000 (Invitrogen). The same reagent was also used to deliver the plasmid vectors to the cells. When cells where transfected with both oligonucleotide(s) and plasmid vectors, the oligonucleotides were always delivered 24 h before the transfection of the cell with plasmids.

### Infectious center assay (ICA) with replication-competent SFV DNA/RNA layered vectors

ICA on the BHK-21 cells was performed as previously described [29,43]. A modified ICA protocol was used to estimate the infectivity of replication-competent DNA/RNA layered vectors for HeLa cells when SFV isolates failed to form distinctive plaques. In this case, the HeLa cells were electroporated with vector DNA using a Bio-Rad (Hercules, CA, USA) Gene Pulser II (one pulse at 220 V/975 μF), collected and suspended in serum free growth medium. Ten-fold serial dilutions of the HeLa cell suspension were prepared in serum-free media and transferred to plates containing 90-100% confluent BHK-21 cells. Their incubation and treatment was carried out as previously described [29].

### RNA purification and reveres-transcription (RT) PCR analysis

Total RNA from transfected or infected cells was purified using TriZol reagent (Invitrogen, USA) and the residual DNA was removed by DNase treatment. cDNA was synthesized using First Strand cDNA synthesis kit (Fermentas; Vilnius, Lithuania), purified and used as a template for PCR amplification with primers SF (5'-TGAAGAAGAGCTGTTTTTACGATCCCTTCA-3') and SR (5'-GGTTGGTACTAGCAACGCACTTTGAATTTTGTAAT-3') and Phusion Hot Start DNA polymerase (Finnzymes, Espo, Finland).

## Results

### Insertion of a thalassemic intron reduces gene expression from an adenovirus vector

Adenoviruses use alternative splicing to express most of their essential genes [14]. Although it is possible to replace the native intron(s) with aberrantly spliced ones, recombinant adenovirus vectors containing a Luc reporter gene with wt or aberrantly spliced introns were constructed to simplify the subsequent analysis. When the pShuttle-CMV plasmid vectors carrying LucWT or Luc705 reporters were assayed in HeLa cells, it was found that the intron with the T705G mutation reduced reporter-gene expression approximately ten-fold (data not shown). The observed reduction was nearly half that previously reported for a very similar assay system [38] and, therefore, a recombinant intron, containing the T705G and C654T mutations, was constructed and inserted into the Luc reporter. It was found that luciferase expression by Luc6+7 was approximately thirty-fold lower than LucWT (data not shown). This result demonstrates the strong inhibitory effect of the artificial intron, probably due to the fact that it possesses two cryptic splicing acceptor sites or because the combination of these sites activates a common cryptic splicing donor site more efficiently than a single cryptic acceptor site. In the context of the recombinant adenovirus infection, however, both aberrantly spliced introns reduced luciferase expression more than fifty-fold (data not shown), indicating that the insertion of a single aberrantly spliced intron efficiently downregulated gene expression from the viral DNA genome.

**Figure 2:**
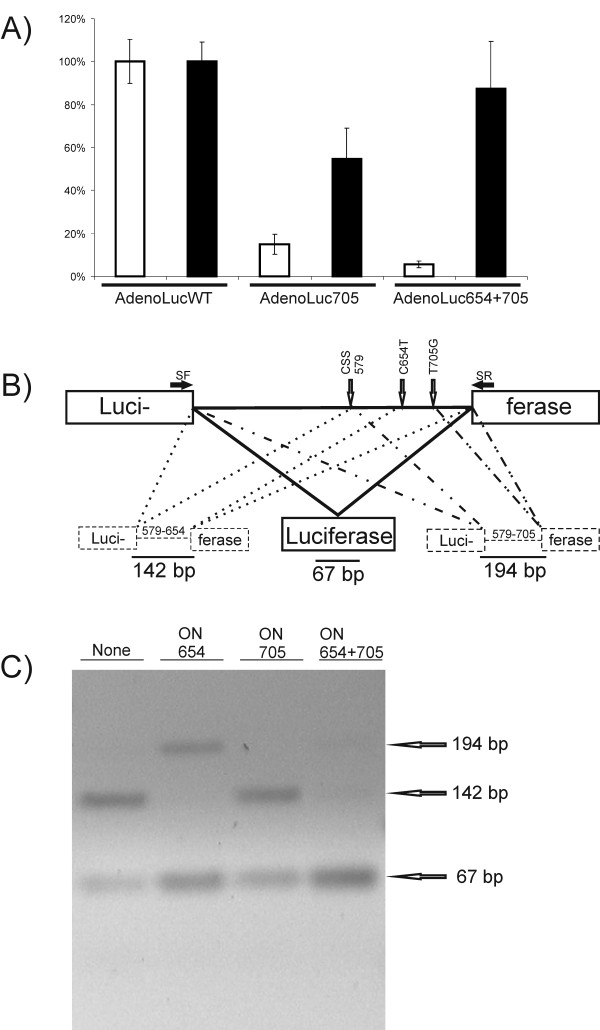
**Effects of insertion of an aberrantly spliced intron and SSOs on adenovirus vector**. A. Expression of luciferase in HeLa cells infected by adenovirus vectors containing wt (AdenoLucWT) or aberrantly spliced (AdenoLuc705 and AdenoLuc654+705) intron in the presence of ONINV (100 nM, open columns) or a mixture of ON654 and ON705 (100 nM, black columns) at 24 h postinfection. The luciferase activities from both series (ONINV and ON654+ON705 transfected cells) were normalized to the activities obtained for AdenoLucWT infected cells, which were defined as 100%, and are represented on the vertical axis. Error bars represent standard deviations; experiments were repeated twice with similar results. B. Schematic presentation of luciferase reporter with recombinant intron and the RT-PCR analysis of splicing products. SF, splice forward primer; SR, splice reverse primer. Positions of thalassemic mutations and common cryptic splice site (CSS) activated by these mutations are shown by open arrows. The aberrant and correct splicing possibilities and mRNAs resulting from these processes are shown, and the length of RT-PCR product is indicated for each splicing variant. C. Agarose-gel electrophoresis analysis of RT-PCR products. SSOs are indicated above the panel; the sizes of expected DNA fragments are shown at the left.

To demonstrate the effects of SSOs, HeLa cells were transfected with negative control oligonucleotide ONINV [42] or a mixture of ON705 and ON654 prior to infection with the recombinant adenovirus. Consistent with previous observations [42], it was found that ONINV slightly activated reporter expression, and we therefore excluded this from further experiments. In contrast, the mixture of SSOs acted as a much more potent activator than ONINV. The expression of the luciferase marker by AdenoLuc705 and AdenoLuc654+705 was activated three-fold and thirteen-fold, respectively (Figure 2A).

To demonstrate that the rescue was indeed caused by splice correction, the mRNAs produced from AdenoLuc654+705 were analyzed by semiquantitative RT-PCR (Figure 2B). This assay revealed that the mRNAs from the Luc6+7 reporter were efficiently spliced because no product corresponding to unspliced RNA was detected (data not shown). In the absence of SSOs, the intron containing two thalassemic mutations was dominantly spliced using the splicing site at position 654. Only small amounts of correctly spliced products were observed and, surprisingly, no use of the incorrect splicing site at position 705 was detected (Figure 2C). Consistently, the presence of ON705 alone had only very small but detectable effect on this splicing pattern, whereas the presence of ON654 moved the splicing site to position 705, causing a concomitant increase in the correctly spliced product (Figure 2C). Finally, the mixture of SSOs almost completely blocked the use of both incorrect splicing sites (Figure 2C). Thus, highly efficient splicing correction was likely the only mechanism behind the observed rescue of luciferase expression, which is consistent with the almost complete recovery of luciferase expression observed in the presence of SSOs (Figure 2A). Consequently, it seems reasonable to assume that gene expression from almost any DNA virus or virus-based vector can be regulated by the insertion of one or more aberrantly spliced introns in concert with splicing correction by their matching SSOs.

### Insertion of aberrantly spliced introns reduces the rescue of replicating RNA from DNA/RNA layered SFV replicon vectors

The sequences of all positive-strand RNA viruses contain numerous motifs corresponding to cryptic splicing sites. In the corresponding DNA/RNA layered expression systems, these cryptic introns have a negative effect on the efficiency of the vector. Such problems could likely be solved by the insertion of efficiently spliced introns into these vectors and/or the removal of cryptic splicing sites by silent mutagenesis [29,31-34]. However, even when introns can be used in DNA/RNA layered vectors, only the rescue of replicating RNA is affected by their presence but not its subsequent replication and transcription.

**Figure 3:**
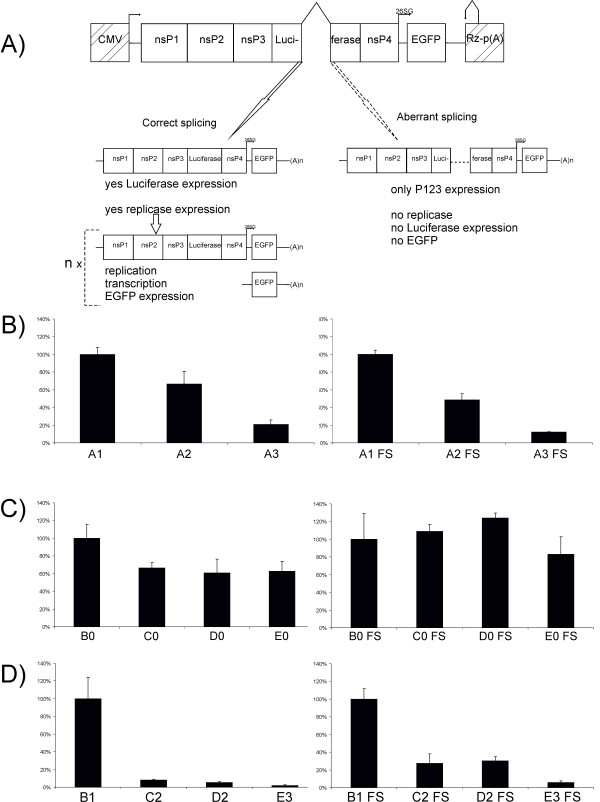
**Effects of insertion of aberrantly spliced introns into SFV DNA/RNA layered replicon vectors**. A. Schematic presentation of vectors A2 and A3 (Table 1). Products of correct splicing (left) represent mRNAs for functional replicase of SFV and for the luciferase marker; the subsequent replication of these RNAs by SFV replicase results in template amplification and transcription of SG-mRNA used for EGFP expression. mRNAs resulting from aberrant splicing (right) express only P123 polyprotein. B. Luciferase activities in HeLa cells at 24 h after transfection with vectors A1, A2 and A3 are represented on the vertical axis; luciferase activities in cells transfected with A1 vectors were taken as 100%. Left panel, results for DNA/RNA layered replicon vectors; right panel, results for DNA/RNA layered replicon vectors containing FS mutation. C. Luciferase activities in HeLa cells at 24 h after transfection with vectors B0, C0, D0 and E0 are represented on the vertical axis; luciferase activities in cells transfected with B0 vectors were taken as 100%. Left panel, results for DNA/RNA layered replicon vectors; right panel, results for DNA/RNA layered replicon vectors containing FS mutation. D. Luciferase activities in HeLa cells at 24 h after transfection with vectors B1, C2, D2 and E3 are represented on the vertical axis; luciferase activities in cells transfected with B1 vectors were taken as 100%. Left panel, results for DNA/RNA layered vectors; right panel, results for DNA/RNA layered vectors containing FS mutation. Error bars on panels B-D represent standard deviations; experiments were repeated three times with similar results.

To demonstrate the effect of an aberrantly spliced intron on the rescue of alphavirus replicons from DNA/RNA layered vectors, we used SFV vectors carrying an EGFP gene under the control of the SG promoter (Figure 1A). In this system, if the introns in LucWT, Luc7 or Luc6+7 reporters are not correctly removed by splicing, the reading frame of the SFV replicase is disrupted. While the luciferase marker can be expressed from correctly spliced transcripts produced by the transcription of the vector plasmid in the cell nucleus and from replicating replicon RNAs produced in the cytoplasm, EGFP expression is here exclusively mediated by viral replicase activity (Figure 3A). Thus, the efficiency of replication can be analyzed by monitoring the expression of these markers. The corresponding assay revealed that the presence of an intron with a T705G mutation in an A2 vector (Table 1) resulted in less than a 50% reduction of luciferase expression compared to the A1 vector, whereas intron with two mutations in an A3 vector resulted in a nearly four-fold decrease of luciferase expression (Figure 3B). The much smaller effect compared to that observed for adenovirus vectors may be caused by the replicative nature of the alphavirus-replicon RNA. Consistent with this concept, it was observed that the EGFP signal appeared, albeit at later time points, in cells transfected with A2 or A3 vectors (data not shown), indicating that the initiation of RNA replication was not blocked, only delayed. To prove that differences in RNA replication were indeed responsible for the diminished difference between the vectors with wt or aberrantly spliced introns, vectors with an FS mutation, preventing expression of functional polymerase and thus any replication/transcription of replicon RNA, were constructed and analyzed. The FS mutation reduced luciferase expression approximately ten-fold (A1FS compared to A1) and, as expected, completely abolished any EGFP expression. A comparison of luciferase expression by these vectors revealed that the intron with the T705G mutation reduced marker expression approximately two-fold and the intron with two mutations reduced marker expression approximately eight-fold (Figure 3B). This indicates that replication is important, but not the sole factor responsible for the relatively small differences in luciferase expression between A1 and the A2 or A3 vectors. Other factors such as the reduced stability of luciferase when expressed in the SFV vector system [44] and/or the toxic effect of nsP2 of SFV on the host cells [45] may also have contributed to this effect.

Next, the effects of an intron introduced into the nsP4 encoding region of the sequence were analyzed. In this case, aberrant splicing was not expected to disrupt luciferase expression from mRNA transcripts made in the nucleus but should still abolish replicase production and, therefore, also the replication and transcription of rescued RNA. It was found that a mutant intron of any kind only caused a minor (approximately 30%) reduction in luciferase expression (Figure 3C). This small effect most probably relates aberrant splicing did not disrupt the luciferase expression and all mRNAs, either made in nucleus or produced by viral replicase complexes, contributed to luciferase expression. Similar to the vectors A2 and A3 the EGFP expression by C0, D0 and E0 vectors was not abolished but slightly delayed. The experiment with corresponding vectors containing FS mutations revealed nearly twenty fold reduction of luciferase expression (B0FS compared to B0) but expectedly failed to reveal any significant effect of the intron in nsP4 region on expression of luciferase (Figure 3C).

Because insertion of one aberrantly spliced intron was clearly insufficient to suppress the release of autonomously replicating transcripts, SFV DNA/RNA layered replicon vectors containing two introns were constructed and analyzed. The results revealed that two introns with T705G mutations or a combination of introns with T705G and C654T mutations resulted in a more than ten-fold reduction of luciferase expression; two introns containing combination of C654T and T705G mutations caused a nearly one-hundred-fold reduction of marker expression (Figure 3D). Thus, combining defective introns resulted in increased inhibition of the rescue of infectious transcripts. The observed inhibitory effects were most significant early after transfection and gradually decreased over time. Coherently, the number of EGFP-positive cells, which was greatly (approximately twenty fold) reduced at the beginning of experiment, gradually increased at the later timepoints. Consistent with the data from previous experiments, it was found that in the context of DNA/RNA layered vectors with an FS mutation, the combination of these introns resulted in inhibitory effects similar to those observed for FS vectors containing a single aberrantly spliced intron in the luciferase-reporter region (compare the right panels of Figure 3B and Figure 3D).

### Insertion of aberrantly spliced introns reduces infectivity of SFV DNA/RNA layered replication-competent vectors

DNA/RNA layered replicon vectors do not produce infectious progeny, and qualitative infectivity assays are thus inapplicable for these vectors. Although the inability to spread greatly increases the safety of these vectors, it also limits their use in many important applications [6]. Therefore, the effects of the insertion of aberrantly spliced introns on the infectivity of DNA/RNA layered replication-competent SFV vectors were analyzed. The vectors used in this study were based on pCMV-SFV4, which contains a wt intron from the rabbit beta-globin gene (Figure 1C). However, it was found that the insertion of one or two wt introns from the human beta-globin gene had no effect on the efficiency of infectious virus rescue from the corresponding constructs (data not shown), indicating that the different introns did not interfere with each other.

**Figure 4:**
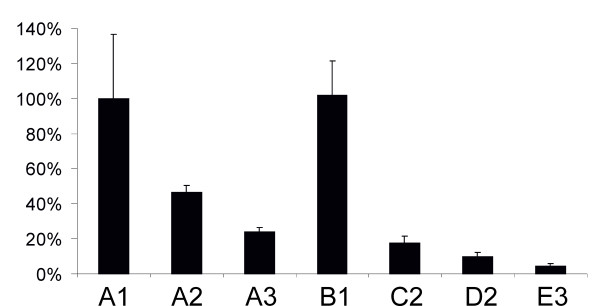
**Effects of insertion of aberrantly spliced introns into SFV DNA/RNA layered replication-competent vectors**. Luciferase activities in HeLa cells at 16 h after transfection with SFV replication-competent DNA/RNA layered vectors containing one (A1, A2 and A3) or two (B1, C2, D2 and E3) introns from the human beta-globin gene are represented on the vertical axis; luciferase activity in cells transfected with A1 vector was taken as 100%. Error bars on represent standard deviations, the results of one reproducible experiment are shown.

Next, a set of DNA/RNA layered replication-competent vectors containing one or two aberrantly spliced introns (Table 1) was constructed and their infectivity was tested using ICA in BHK-21 cells. The results of this analysis (Table 2) showed that the insertion of an intron with a T705G mutation into the luciferase reporter region reduced the infectivity of the vector by 50%, while the insertion of an intron with two mutations resulted in a nearly five-fold drop in infectivity. The insertion of two aberrantly spliced introns caused more pronounced effects, ranging from ten-fold reductions for the C2 and D2 vectors to a fifty-fold reduction for the E3 vector. When ICA was performed with HeLa cells, only the constructs lacking an aberrantly spliced intron produced plaques (Table 2). Taking into account the detection limit of this experiment, we concluded that the effect of an aberrantly spliced intron or introns was greater in HeLa than in BHK-21 cells. This may result from the fact that the introns with the IVS2^705 ^and IVS2^654 ^mutations had a human origin and therefore the mutations had less effect in rodent cells. Alternatively, the difference may originate from the different efficiency of the alphavirus DNA/RNA layered vector in different cell lines, as previously described for DNA/RNA layered vectors derived from the Sindbis virus [46]. Nonetheless, the infectivity of the DNA/RNA layered replication-competent vectors in BHK-21 cells was in accord with the luciferase expression by DNA/RNA layered replicon vectors with similar designs in HeLa cells (Table 2; Figure 3B, D). To test whether this correlation also existed for replication-competent vectors, the corresponding vectors were transfected into HeLa cells and the luciferase activity was measured 16 h later. The results of this analysis (Figure 4) were consistent with those obtained using ICA; however, the correlation was lost when luciferase activities were measured 30 h or more post-transfection (data not shown). The presence of a single aberrantly spliced intron inside the luciferase reporter region caused three- (A2) to five-fold (A3) reductions in luciferase expression, while the combination of two aberrantly spliced introns caused a six- (C2, D2 vectors) to more than one-hundred-fold (E3) effect (Figure 4). Thus, luciferase activity can also be used to accurately estimate the infectivity of the corresponding DNA/RNA layered replication-competent vectors.

**Table 2:** Infectivity of SFV replication-competent vectors in BHK-21 and HeLa cells (in PFU/pmol of vector DNA).

ell line Construct	BHK-21	HeLa
A1	1.3 × 10^6^	3.6 × 10^4^

A2	7.6 × 10^5^	Nd

A3	2.8 × 10^5^	Nd

B1	1.0 × 10^6^	2.4 × 10^4^

C2	1.1 × 10^5^	Nd

D2	1.0 × 10^5^	Nd

E3	2.5 × 10^4^	Nd

nd: not detected (below 100 PFU/pmol).

### Rescue of the activity of DNA/RNA replicon and replication competent vectors by SSOs

**Figure 5:**
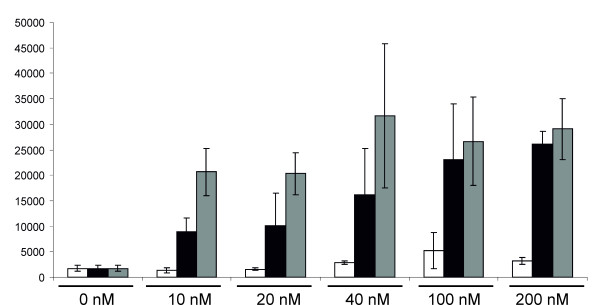
**Rescue of the defect caused by insertion of an aberrantly spliced intron**. Rescue of the defect caused by insertion of an aberrantly spliced intron into DNA/RNA layered replicon vectors by ON654, ON705 or their combination. HeLa cells were transfected with the indicated SSOs at final concentrations of 0, 10, 20, 40, 100 or 200 nM 24 h prior to transfection with E3 vector. Luciferase activity (in relative luciferase units) produced by the vector at 24 h post-transfection is represented on the vertical axis. Open columns represent activities obtained in the presence of ON705, gray columns represent activities obtained in the presence of ON654 and black columns represent activities obtained in the presence of both SSOs. Error bars represent standard deviations; the results of one of three reproducible experiments are shown.

The effect caused by the insertion of aberrantly spliced introns can be reversed through the use of SSOs with plasmid vectors in transfected cells or in stable cell lines [38] and with recombinant adenovirus vectors (Figure 2A). The reversion depends on the concentration of SSOs [38] and can be greatly enhanced by applying oligonucleotides with splice-enhancing activities [40,47]. To determine whether this was also the case for SFV DNA/RNA layered replicon vectors, the activity of an E3 replicon was analyzed in the presence of ON705 or/and ON654. Despite the presence of target sites for both SSOs, they were found to be unequally efficient. ON705 had, even at concentration of 100 nM, only a modest effect on the rescue of self-replicating transcripts, whereas in the presence of ON654, activity was observed rapidly, even at a concentration of 10 nM (Figure 5). When the SSOs were combined, a synergistic effect was observed. The rescue was already efficient at 10 nM; it peaked at a 40 nM concentration and did not change significantly at higher concentrations (Figure 5). RT-PCR analysis revealed that the splicing pattern of the intron in the E3 vector, as well as the effects of SSOs on this pattern, were identical to those observed with an adenovirus vector (data not shown). This explains, at least in part, the more prominent effect of ON654 and suggests that ON654 has some splice-enhancing activity, even though its target site does not overlap with the target of the previously characterized splice-enhancing SSO [47]. Thus, both SSOs were needed to reverse the effects caused by a combination of T705G and C654T mutations, and ON654 clearly had the greater contribution to this rescue effect.

**Figure 6:**
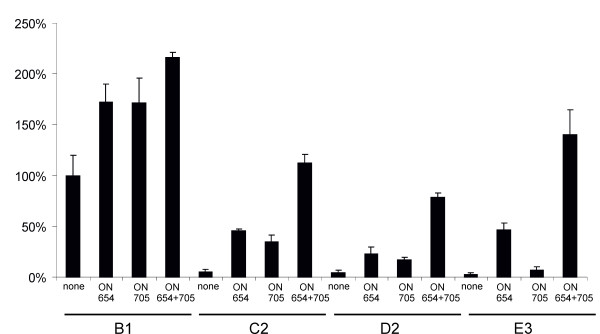
**Rescue of the activity of the SFV DNA/RNA layered replication-competent vectors by SSOs**. HeLa cells were mock transfected (control cells, indicated as "none") or transfected with SSOs (ON654, ON705 or their combination) at a final concentration 100 nM 24 hours prior to transfection with vectors B1, C2, D2 or E3. Infectivity of constructs was indirectly estimated by measuring the luciferase activity at 16 h post-transfection; luciferase activities, normalized to that in cells transfected with B1 vector in the absence of SSOs (100%), are represented on the vertical axis. Error bars represent standard deviations; the results of one of three reproducible experiments are shown

Next, it was confirmed that SSOs were also capable of rescuing the activity of DNA/RNA layered replication-competent SFV vectors containing one (data not shown) or two aberrantly spliced introns (Figure 6). The presence of these SSOs or their combination resulted in up to a two-fold activation of rescue and/or subsequent replication of the B1 vector containing only wt introns. A similar enhancement effect on SFV infection was often observed when infected cells were treated with Lipofectamine 2000 and scrambled oligonucleotides (our unpublished data). This effect may be caused by changes in the lipid composition of the plasma membranes and/or endosomal membranes used for SFV replication [19] due to lipids included in the transfection reagent. The effects of SSOs on the DNA/RNA layered replication-competent vectors containing aberrantly spliced introns were, however, much more prominent. Again, ON654 invariably mediated greater activation than did ON705 (Figure 6), even for the D2-vector bearing only T705G mutations. Consistent with prior results, the rescue was most efficient when both SSOs were applied. The magnitude of activation was over twenty times for the D2 and C2 vectors (Figure 6), clearly indicating that most of it originated from splice correction. The biggest activation (over one-hundred-fold) was observed for the E3 vector, where its activity, which in the absence of SSOs was almost at a background level, was restored to a level comparable to that of the B1 vector. These results imply that mutants with even greater defects, for example a larger number of defective introns, can probably be efficiently rescued with an appropriate mixture of SSOs.

## Discussion

The basics of splice-switch technology were developed nearly two decades ago and since then the technology and its variants, such as exon skipping, have been envisioned as potent approaches to the treatment of several genetic disorders [25,48], inflammation [27] and cancer [26]. The great potential of this technology, especially evident after the first successes in *in vivo *applications, naturally raised the question of whether the splice-switch platform is also applicable for viruses and virus-based vectors. One possible application would be to use it against viruses that use splicing. In this case, the SSOs can act as antiviral agents by causing deregulation of viral gene expression and subsequently block viral infection. This method may have potential as a new therapeutic approach against viruses that are difficult to target by conventional means, for example, those using latent infection accompanied with complicated splicing and minimal protein expression. However, in general, this method would be unlikely to have advantages over the inhibition of viral gene expression and replication using conventional antisense oligonucleotides or siRNAs that target viral RNAs for degradation or block the expression of viral proteins [49].

An alternative possibility is to use splice-switch technology to control the release, gene expression, replication and virus-induced pathologies for genetically modified viruses, as demonstrated in this study for the adenovirus vector and layered DNA/RNA SFV vectors. Due to the different nature of the systems used, the impact of splice-switching would be different in each. When applied to an adenovirus, splice switching generates permanent control over viral infection, while in the case of DNA/RNA layered vectors, only the release of self-replicating RNA genomes or replicons can be controlled. Indeed, it was found that the introduction of aberrantly spliced introns into a gene-expression unit of a recombinant adenovirus had a stronger effect (Figure 2A) than a similar insertion made into alphavirus layered DNA/RNA vectors (Figure 3B, C, 4). The observed effect was, especially at later time points, completely lost for replication-competent SFV due to the subsequent round of infection (data not shown). Thus, the insertion of a single aberrantly spliced intron was insufficient to suppress the release of infectious replicon RNAs or viral genomes from DNA/RNA layered vectors. Therefore, it is important that the insertion of two aberrantly spliced introns into DNA/RNA layered vectors of SFV resulted in increased effect and efficiently suppressed the release of infectious RNA (Figure 3D, 4).

The infection of cells by an alphavirus can be initiated by single copy of correctly delivered and released genomic RNA. When the *in vitro *transcribed RNA is delivered by transfection, the efficiency is much lower (typically ~10^7 ^pfu/pmol of RNA), possibly reflecting the fact that transfection is not nearly as effective as natural RNA delivery in infection. That efficiency is often further reduced by approximately one log for DNA/RNA layered vectors, probably because of the necessity to transport the DNA into the nucleus of the cell and to export produced RNA from the nucleus to the cytoplasm where it can initiate replication. Indeed, it has been demonstrated by the use of stable cell lines containing integrated copies of DNA/RNA layered vectors that the nuclear export and stability of produced RNA are likely the critical steps limiting the efficiency of DNA/RNA layered vectors [46]. It has also been suggested that RNAs made in the nucleus are intrinsically less efficient in replication initiation than those delivered by a natural infection route: single-copy transcript made in the nucleus is generally insufficient to initiate replication, instead the presence of a minimal threshold level of RNA transcripts is required [46]. RT-PCR analysis of splicing products revealed that even in the case of the "strongest" aberrantly spliced intron, a considerable amount of correctly spliced products were present, corresponding to the potentially self-replicating replicon RNAs. Additionally, the appearance of EGFP in cells transfected with such DNA/RNA layered replicon vectors was not blocked but delayed. Both of these facts are consistent with the model suggested by Boorsma et al., 2003 [46]. Additionally, the threshold level itself may be higher for DNA/RNA layered vectors containing aberrant intron(s). Data obtained for vectors with FS mutations indicates that aberrant splicing did not negatively affect the transport of mRNA from the nucleus or its stability (Figure 3C). Thus, these mRNAs, which were not capable of producing a functional replicase (Figure 3A) but did contain the correct *cis*-elements required for replication and transcription, accumulated in transfected cells. It is also known that the alphavirus replicase complex, when provided *in trans*, is capable of replicating such templates [18,22] and, therefore, these RNA molecules may act as defective interfering genomes. The nsP1-3 proteins of SFV and their precursors are also translated from aberrantly-spliced RNAs and may have effect on the the formation of functional replicase complexes. Combined with their reduced copy number, this would likely result in a situation in which the threshold levels of correctly spliced RNAs are reached at later times. Additionally, the time taken to reach required threshold levels is limited by the loss or inactivation of vector DNA or by the death or severe damage of transfected cells due to the toxic effects of nsP2 proteins [45] translated from defective RNAs (Figure 3A). Consequently, the required RNA threshold levels may not be reached at all, as was likely the case for the HeLa cells transfected with replication-competent DNA/RNA layered vectors (Table 2). Although our data indicates that the insertion of a larger number of aberrantly spliced introns will further enhance the suppression of infectious RNA release, there may be no need to block it completely. A reduction in the rate of accumulation of correctly spliced RNAs is likely sufficient to block the release of infectious viruses.

Despite the fact that inhibition of adenovirus vector gene expression by the insertion of aberrantly spliced introns was straightforward and multiple factors contributed to reduce the release of infectious alphavirus replicons or genomes, both systems could be efficiently rescued by SSOs. Interestingly, the rescue by the combination of two SSOs was always nearly complete, and, surprisingly, more significant defects caused by aberrantly spliced intron(s) led to higher rescue efficiencies (Figure 2A, C; Figure 6). Because the rescue rate for the E3 vector (Figure 5) and D2 vector (Figure 6) by ON705 alone was low, we concluded that the presence of SSOs with splicing-enhancement activity is required to achieve high-level or even complete reversion of defects caused by the insertion of aberrantly spliced introns.

## Conclusions

The results of this study show that splice-switching is feasible for the construction of conditionally activated systems of different viruses and could be applicable in vaccination or virus-based gene or anticancer therapy. The method is straightforward and can be easily combined with other techniques used for the construction of regulated viral vectors. Unlike most manipulations made at the level of the replicating genome, the inhibition of infectious genome rescue cannot be corrected or compensated by activity of non-proofreading viral RNA replicase. Thus, the splice-switch technology can be used to achieve a targeted rescue of the viral genome from the vectors in cells where SSOs are present. However, as SSOs cannot affect the subsequent spread of alphavirus, additional control measures may be required, such as the insertion of suicide genes [50]. The possibilities for practical application of this approach depend on the development and improvement of methods for tissue- and cell-type-specific delivery of SSOs, which act as essential cofactors for the release and (in the case of DNA viruses) propagation of such viral vectors.

## Competing interests

LV, GH, TL, SELA, UL and AM are also co-inventors in patent application P200900077 "A method and composition for creating conditional lethality in virus mutants and for eliminating the viability of a eukaryotic cell", which describes potential industrial applications of several vectors and approaches described in this manuscript; the owner of IP rights is the University of Tartu.

## Authors' contributions

LV performed experiments with replication-competent virus vectors, GH performed experiments with adenovirus and SFV replicon vectors. KP and TL participated in experiments with SSOs, SELA and ÜL developed the concept and designed approaches, AM designed experiments, supervised the project and wrote the manuscript. All authors read and approved the final manuscript.

## References

[B1] Toth K Dhar D Wold WS Oncolytic (replication competent) adenoviruses as anti-cancer agents Expert Opin Biol Ther 2010 10 353 368 10.1517/14712590903559822 20132057

[B2] Kanai R Wakimoto H Cheema T Rabkin SD Oncolytic herpes simplex virus vectors and chemotherapy: are combinatorial strategies more effective for cancer? Future Oncol 2010 6 619 634 10.2217/fon.10.18 20373873 PMC2904234

[B3] Rommelaere J Geletneky K Angelova AL Daeffler L Dinsart C Kiprianova I Schlehofer RJ Raykov Z Oncolytic parvoviruses as cancer therapeutics Cytokine Growth Factor Rev 2010 21 185 195 10.1016/j.cytogfr.2010.02.011 20211577

[B4] Harrington KJ Karapanagiotou EM Roulstone V Twigger KR White CL Vidal L Beirne D Prestwich R Newbold K Ahmed M Thway K Nutting CM Coffey M Harris D Vile RG Pandha HS Debono JS Melcher AA Two-stage phase I dose-escalation study of intratumoral reovirus type 3 dearing and palliative radiotherapy in patients with advanced cancers Clin Cancer Res 2010 16 3067 3077 10.1158/1078-0432.CCR-10-0054 20484020 PMC3907942

[B5] Barber GN Vesicular stomatitis virus as an oncloytic vector Viral Immunol 2004 17 516 527 10.1089/vim.2004.17.516 15671748

[B6] Atkins GJ Fleeton MN Sheahan GJ Therapeutic and prophylactic applications of alphavirus vectors Expert Rev Mol Med 2008 11 33 10.1017/S1462399408000859 19000329

[B7] Heise C Sampson-Johannes A Williams A McCormick F Von Hoff DD Kirn DH ONYX-015, an E1B gene-attenuated adenovirus, causes tumor-specific cytolysis and antitumoral efficacy that can be augmented by standard chemotherapeutic agents Nat Med 1997 3 639 645 10.1038/nm0697-639 9176490

[B8] Breidenbach M Rein TD Wang M Nettelbeck DM Hemminki A Ulasov I Rivera AR Everts M Alvarez RD Douglas JT Curiel DT Genetic replacement of adenovirus shaft fiber reduces liver tropism in ovarian cancer gene therapy Hum Gene Ther 2004 15 509 518 10.1089/10430340460745829 15144580

[B9] Myhre S Henning P Freidman M Stahl S Lindholm L Magnusson MK Re-targeted adenovirus vectors with dual specificity; binding specificities conferred by two different Affibody molecules in fiber Gene Ther 2009 16 252 261 10.1038/gt.2008.160 18946496

[B10] Latham JP Searle PF Mautner V James ND Prostate-specific antigene promoter/enhancer driven gene therapy for prostate cancer: construction and testing of tissue-specific adenovirus vector Cancer Res 2000 60 334 341 10667585

[B11] Wang ZX Bian HB Yang JS De W Ji XH Adenovirus-mediated suicide gene therapy under the control of Cox-2 promoter for colorectal cancer Cancer Biol Ther 2009 8 1480 1488 10.4161/cbt.8.15.8940 19571664

[B12] Edge RE Falls TJ Brown CW Lichty BD Atkins H Bell JC A let-7 MicroRNA-sensitive vesicular stomatitis virus demonstrates tumor-specific replication Mol Ther 2008 16 1437 1443 10.1038/mt.2008.130 18560417

[B13] Gomez-Manzano C Fueyo J Oncolytic adenoviruses for treatment of brain tumors Curr Opin Mol Ther 2010 12 530 537 20886384

[B14] Berk AJ Knipe DM, Howley PM Adenoviridae: the viruses and their replication Fields Virology 2007 2 fifth Lippincott Williams & Wilkins/Wolters Kulwer, Philadelphia 2355 2394

[B15] Jones N Shenk T An adenovirus type 5 early gene function regulates expression of other early viral genes Proc Natl Acad Sci USA 1979 76 3665 3669 10.1073/pnas.76.8.3665 291030 PMC383893

[B16] Hearing P Shenk T The adenovirus type 5 E1A transcriptional control region contains a duplicated enhancer element Cell 1983 33 695 703 10.1016/0092-8674(83)90012-0 6871991

[B17] He TC Zhou S da Costa LT Yu J Kinzler KW Vogelstein B A simplified system for generating recombinant adenoviruses Proc Natl Acad Sci USA 1998 95 2509 2514 10.1073/pnas.95.5.2509 9482916 PMC19394

[B18] Frolova EI Gorchakov R Pereboeva L Atasheva S Frolov I Functional Sindbis virus replicative complexes are formed at the plasma membrane J Virol 2010 84 11679 11695 10.1128/JVI.01441-10 20826696 PMC2977861

[B19] Spuul P Balistreri G Kääriäinen L Ahola T Phosphatidylinositol 3-kinase-, actin-, and microtubule-dependent transport of Semliki Forest Virus replication complexes from plasma membrane to modified lysosomes J Virol 2010 84 7543 7557 10.1128/JVI.00477-10 20484502 PMC2897599

[B20] Kääriäinen L Ahola T Functions of alphavirus nonstructural proteins in RNA replication Prog Nucleic Acid Res Mo?? Biol 2002 71 187 222 10.1016/S0079-6603(02)71044-1 PMC7133189 12102555

[B21] Rausalu K Iofik A Ülper L Karo-Astover L Lulla V Merits A Properties and use of novel replication-competent vectors based on Semliki Forest virus Virol J 2009 6 33 10.1186/1743-422X-6-33 19317912 PMC2669057

[B22] Liljeström P Garoff H A new generation of animal cell expression vectors based on the Semliki Forest virus replicon Biotechnology (N Y) 1991 9 1356 1361 10.1038/nbt1291-1356 1370252

[B23] Liljeström P Lusa S Huylebroeck D Garoff H In vitro mutagenesis of a full-length cDNA clone of Semliki Forest virus: the small 6,000-molecular-weight membrane protein modulates virus release J Virol 1991 65 4107 4113 2072446 10.1128/jvi.65.8.4107-4113.1991 PMC248843

[B24] Dubensky TW Jr Driver DA Polo JM Belli BA Latham EM Ibanez CE Chada S Brumm D Banks TA Mento SJ Jolly DJ Chang SMW Sindbis virus DNA-based expression vectors: utility for in vitro and in vivo gene transfer J Virol 1996 70 508 519 8523564 10.1128/jvi.70.1.508-519.1996 PMC189839

[B25] Bauman J Jearawiriyapaisarn N Kole R Therapeutic potential of splice-switching oligonucleotides Oligonucleotides 2009 19 1 13 10.1089/oli.2008.0161 19125639 PMC2663420

[B26] Bauman JA Li SD Yang A Huang L Kole R Anti-tumor activity of splice-switching oligonucleotides Nucl Acids Res 2010 38 8348 8356 10.1093/nar/gkq731 20719743 PMC3001088

[B27] Graziewicz MA Tarrant TK Buckley B Roberts J Fulton L Hansen H Ørum H Kole R Sazani P An endogenous TNF-alpha antagonist induced by splice-switching oligonucleotides reduces inflammation in hepatitis and arthritis mouse models Mol Ther 2008 16 1316 1322 10.1038/mt.2008.85 18461057 PMC2671678

[B28] van Ommen GJ van Deutekom J Aartsma-Rus A The therapeutic potential of antisense-mediated exon skipping Curr Opin Mol Ther 2008 10 140 149 18386226

[B29] Ülper L Sarand I Rausalu K Merits A Construction, properties, and potential application of infectious plasmids containing Semliki Forest virus full-length cDNA with an inserted intron J Virol Methods 2008 148 265 270 10.1016/j.jviromet.2007.10.007 18054090 PMC7172237

[B30] Johansen IE Intron insertion facilitates amplification of clones virus cDNA in *Escherichia coli *while biological activity is re-established after transcription in vivo Proc Natl Acad Sci USA 1996 93 12400 12405 10.1073/pnas.93.22.12400 8901593 PMC38003

[B31] Yang SJ Revers F Souche S Lot H Le Gall O Candresse T Dunez J Construction of full-length cDNA clones of lettuce mosaic virus (LMV) and the effects of intron-insertions on their viability in *Escherichia coli *and their infectivity to plants Arch Virol 1998 143 2443 2451 10.1007/s007050050474 9930200

[B32] Lopez-Moya JJ Garcia JA Construction of a stable and highly infectious intron-containing cDNA clone of plum pox potyvirus and its use to infect plants by particle bombardment Virus Res 2000 68 99 107 10.1016/S0168-1702(00)00161-1 10958981

[B33] Marillonnet S Thoeringer C Kandzia R Klimyuk V Gleba Y Systemic Agrobacterium tumifaciens-mediated transfection of viral replicons for efficient transient expression in plants Nature Biotechnol 2005 23 718 723 10.1038/nbt1094 15883585

[B34] Gonzalez JM Penzes Z Almazan F Calvo E Enjuanes L Stabilization of a full-length infectious cDNA clone of transmissible gastroenterits coronavirus by insertion an intron J Virol 2002 76 4644 4661 10.1128/JVI.76.9.4655-4661.2002 PMC155106 11932433

[B35] Busslinger M Moschonas N Flavell RA Beta+thalassemia: aberrant splicing results from a single point mutation in an intron Cell 1981 27 289 298 10.1016/0092-8674(81)90412-8 6895866

[B36] Jo T Momita S Sadamori N Tomonaga M Fucharoem S Fukumaki Y Ichimaru M A case of theta-thalassemia with a C----T substitution at position 654 of the second intervening sequence of the beta-globin gene Intern Med 1992 31 269 272 10.2169/internalmedicine.31.269 1600278

[B37] Dominski Z Kole R Restoration of correct splicing in thalassemic pre-mRNA by antisense oligonucleotides Proc Natl Acad Sci USA 1993 90 8673 8677 10.1073/pnas.90.18.8673 8378346 PMC47420

[B38] Kang SH Cho MJ Kole R Up-regulation of luciferase gene expression with antisense oligonucleotides: implications and applications in functional assay development Biochemistry 1998 37 6235 6239 10.1021/bi980300h 9572837

[B39] Roberts J Palma E Sazani P Ørum H Cho M Kole R Efficient and persistent splice switching by systemically delivered LNA oligonucleotides in mice Mol Ther 2006 14 471 475 10.1016/j.ymthe.2006.05.017 16854630

[B40] Svasti S Suwanmanee T Fucharoen S Moulton HM Nelson MH Maeda N Smithies O Kole R RNA repair restores hemoglobin expression in IVS2-641 thalassemic mice Proc Natl Acad Sci USA 2009 106 1205 1210 10.1073/pnas.0812436106 19164558 PMC2633555

[B41] Tamberg N Lulla V Fragkoudis R Lulla A Fazakerley JK Merits A Insertion of EGFP into the replicase gene of Semliki Forest virus results in a novel, genetically stable marker virus J Gen Virol 2007 88 1225 1230 10.1099/vir.0.82436-0 17374766 PMC2274952

[B42] Guterstam P Lindgren M Johansson H Tedebark U Wangel J El Andaloussi S Langel Ü Splice-switching efficiency and specificity for oligonucleotides with locked nucleic acid monomers Biochem J 2008 412 307 313 10.1042/BJ20080013 18271753

[B43] Gorchakov R Frolova E Williams BR Rice CM Frolov I PKR-dependent and -independent mechanisms are involved in translational shutoff during Sindbis virus infection J Virol 2004 78 8455 8467 10.1128/JVI.78.16.8455-8467.2004 15280454 PMC479073

[B44] Varjak M Žusinaite E Merits A Novel functions of the alphavirus non-structural protein nsP3 C-terminal region J Virol 2010 84 2352 2364 10.1128/JVI.01540-09 20015978 PMC2820926

[B45] Garmashova N Gorchakov R Frolova E Frolov I Sindbis virus nonstructural protein nsP2 is cytotoxic and inhibits cellular transcription J Virol 2006 80 5686 5696 10.1128/JVI.02739-05 16731907 PMC1472573

[B46] Boorsma M Saudan P Pfruender H Bailey JE Schlesinger S Renner WA Bachmann FM Alphavirus cDNA-based expression vectors: effects of RNA transcription and nuclear export Biotechnol Bioeng 2003 81 553 562 10.1002/bit.10496 12514804

[B47] Resina S Kole R Travo A Lebleu B Thierry AR Switching on transgene expression by correcting aberrant splicing using multi-targeting steric-blocking oligonucleotides J Gen Med 2007 9 498 510 10.1002/jgm.1044 17471591

[B48] Lu QL Yokota T Takeda S Garcia L Muntoni F Partridge T The status of exon skipping as therapeutic approach to Duchenne muscular dystrophy Mol Ther 2011 19 9 15 10.1038/mt.2010.219 20978473 PMC3017449

[B49] Haasnoot J Berkhout B Nucleic acid-based therapeutics in the battle against pathogenic viruses Handb Exp Pharmacol 2009 189 243 263 10.1007/978-3-540-79086-0_9 19048203 PMC7119910

[B50] Tseng JC Daniels G Meruelo D Controlled propagation of replication-competent Sindbis viral vector using suicide gene strategy Gene Ther 2009 16 291 296 10.1038/gt.2008.153 18818670

